# *Pasteurella canis* infection caused by a dog bite leads to osteomyelitis and genomic analysis of the isolate

**DOI:** 10.1002/jcla.23274

**Published:** 2020-03-02

**Authors:** Zhongliang Zhu, Jiawen Lu, Ying Chen, Fang He

**Affiliations:** ^1^ Department of Clinical Laboratory Zhejiang Provincial People's Hospital People’s Hospital of Hangzhou Medical College Hangzhou China

Dear Editor,

*Pasteurella canis* is a gram‐negative coccobacillus mainly resides in the oral cavity, nasopharynx, or intestine of domestic animals.^1^ It is an important bacterial pathogen in both animals and humans.^2^ *P. canis* can cause a variety of infections that lead to osteomyelitis, keratitis, peritonitis, and bacteraemia.^3^, ^4^, ^5^, ^6^ In humans, dog bites are a potential risk of infection caused by this pathogen.^5^ There are reports of *P. canis* infection in humans following a scratch or bite from a domestic animal.^7^ These studies have been carried out mainly on the phenotype of the bacteria, with little genomic research.^8^ The genomic characteristics of this bacterial pathogen still need to be better elucidated. Here, we report the first genome sequence of a clinical *P. canis* strain isolated from a patient diagnosed with index finger osteomyelitis in China. Genotypic characterization of this strain was further analyzed.

A 76‐year‐old male patient was diagnosed with index finger osteomyelitis and hospitalized in the department of hand surgery in a teaching hospital in Zhejiang Province in August 2019. This patient was accidentally bitten by a dog, which injured his right hand and led to bleeding and pain, 20 days prior to his hospitalization. He immediately went to the community hospital for treatment, which included debridement, suture bandaging, and rabies vaccine injection. Ten days prior to his hospitalization, his right hand showed redness, swelling, pain, and suppuration. The patient used to be physically fit. There was no history of diabetes, coronary heart disease, or infectious disease except for hypertension. The results of initial routine blood tests were as follows: white blood cells (WBCs), 9.41 × 10^9^/L (neutrophils 73.9% and lymphocytes 18.2%); platelets, 228 × 10^9^/L; and hemoglobin, 148 g/L. The patient underwent two rounds of debridement of osteomyelitis while in the hospital. The patient's right index finger was infected from the distal segment to the proximal segment with tendon infection, tendon necrosis, tendon sheath infection, soft tissue infection, and bone defect. The wound was thoroughly debrided. Necrotic bone, tendons, and necrotic tissue were all removed. Cefuroxime sodium was used for antibacterial treatment after surgery. Bacterial culture of pus samples suggested a *P. canis* infection.

The isolate was preliminarily identified using the VITEK MS system (bioMérieux, France) and was further confirmed by calculating the average nucleotide identity (ANI), one of the bacterial whole‐genome similarity metrics. The ANI results revealed that the genome of *P. canis* strain QBSD was over 99.89% identical to the type strain *P. canis* NCTC11621. Antimicrobial susceptibility testing was conducted using the Etest method following the guidelines of the Clinical and Laboratory Standards Institute (CLSI). Piperacillin, ceftazidime, ceftriaxone, cefepime, imipenem, levofloxacin, and tetracycline were used in the test. The whole‐genome sequence of the strain was determined using the Illumina NovaSeq 6000 platform (Illumina Inc). The short reads generated were de novo assembled into contigs using SPAdes 3.13.0. The whole‐genome sequence was automatically annotated by the NCBI Prokaryotic Genome Annotation Pipeline (PGAP) server. Antimicrobial resistance genes, virulence genes, and plasmid replicons of the isolate were analyzed using the BacWGSTdb server.^9^, ^10^

The whole‐genome sequence of *P. canis* strain QBSD consisted of 30 contigs that comprised 2 231 959 bp, and the PGAP server predicted a total of 2033 protein‐coding sequences. The overall G + C content of this isolate amounted to 36.7%. In total, 52 tRNA genes, 11 rRNA genes, and 4 ncRNA operons were identified. The genome contained several IS elements, the majority of which belong to the IS*1595*, IS*3,* and IS*200* families. Two confirmed CRISPR sequences and one putative secondary metabolite gene cluster bacteriocin could also be predicted. The antimicrobial resistance genes, virulence genes, and plasmid replicons of *P. canis* strain QBSD are presented in Table 1. One quinolone resistance gene, *qnrS1*, and one plasmid replicon, ColRNAI, could be identified in the genome. Five virulence genes were identified in *P. canis* strain QBSD, which were *gmhA/lpcA*, *hitA*, *inv*, *kdsA,* and *lpxC*.

**Table 1:** Antimicrobial resistance genes, virulence genes, and plasmid replicons in *Pasteurella canis* strain QBSD

Antimicrobial resistance gene	Contig	Identity (%)	Position	Antimicrobial resistance category
*qnrS1*	contig00021	99.85	454…1110	quinolone

Virulence gene	Contig	Identity (%)	Position	Functional annotation
*gmhA/lpcA*	contig00003	85.32	175 625…176 135	Phosphoheptose isomerase
*hitA*	contig00002	83.65	259 731…260 562	Periplasmic iron‐binding protein
*inv*	contig00024	93.06	1037…1180	Invasin
*kdsA*	contig00001	83.17	96 418…97 148	2‐dehydro‐3‐deoxyphosphooctonate aldolase
*lpxC*	contig00003	82.18	198 819…199 688	UDP‐3‐O‐(R‐3‐hydroxymyristoyl)‐N‐acetylglucosamine deacetylase

Plasmid replicon	Contig	Identity (%)	Position	Annotation
ColRNAI	contig00019	92.23	1062…1164	Gram‐negative

A total of two *P. canis* strains could be found in the NCBI GenBank database. Orthologous genes between QBSD and these two strains were identified using Roary and OrthoVenn, and phylogenetic relationships were determined by NJ/UPGMA phylogeny based on core genome single nucleotide polymorphism analysis. Comparative genomic analyses of the three *P. canis* isolates revealed that they shared a large number of genes. However, phylogenetic analysis showed that these strains were not epidemiologically related (Figure 1).

**Figure 1:**
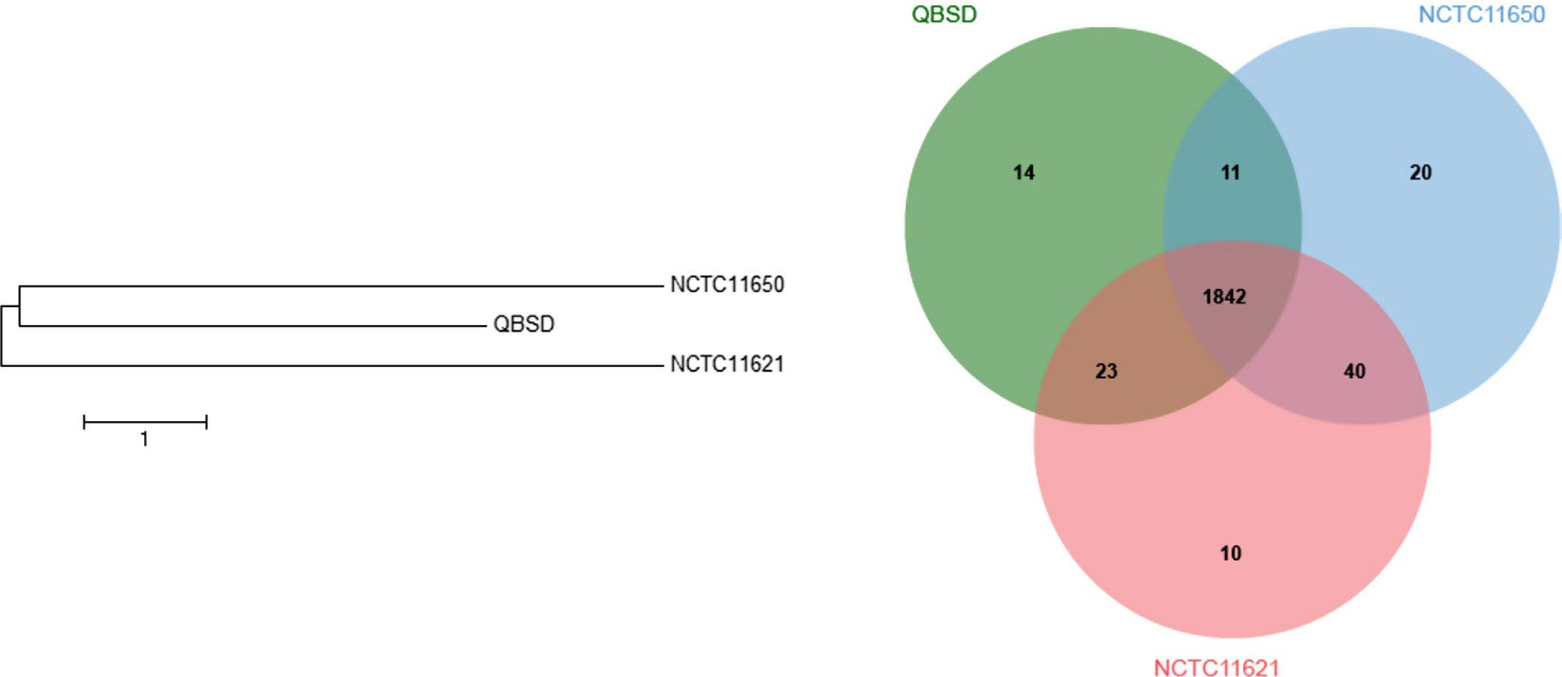
Phylogenetic relationship between QBSD and a total of two *Pasteurella canis* strains deposited in the NCBI GenBank database (*P. canis* strain NCTC11621, accession number UGTV01; *P. canis* strain NCTC11650, accession number UATN01). Venn diagram showing the orthologous groups in the three *P. canis* isolates. Numbers inside the Venn diagrams indicate the number of genes found to be shared among the given genomes

In summary, we report the first genome sequence of a clinical *P. canis* strain isolated from a patient diagnosed with index finger osteomyelitis in China. Our data may help to understand the genomic features of this bacterial pathogen.

This Whole Genome Shotgun project has been deposited at DDBJ/EMBL/GenBank under the accession number WUMP00000000.

## References

[1] Yefet E , Abozaid S , Nasser W , Peretz A , Zarfin Y . Unusual infection–Pasteurella canis bacteremia in a child after exposure to rabbit secretions. Harefuah. 2011;150(1):13‐15, 70. 21449149

[2] Hazelton BJ , Axt MW , Jones CA . Pasteurella canis osteoarticular infections in childhood: review of bone and joint infections due to pasteurella species over 10 years at a tertiary pediatric hospital and in the literature. J Pediatr Orthop. 2013;33(3):e34‐e38. 23482278 10.1097/BPO.0b013e318287ffe6

[3] Shah A , Talati M , Mauger T . Medical and surgical management of Pasteurella canis infectious keratitis. IDCases. 2017;9:42‐44. 28660128 10.1016/j.idcr.2017.05.012 PMC5479940

[4] Albert TJ , Stevens DL . The first case of Pasteurella canis bacteremia: a cirrhotic patient with an open leg wound. Infection. 2010;38(6):483‐485. 20623245 10.1007/s15010-010-0040-1

[5] Kim B , Pai H , Lee KH , Lee Y . Identification of pasteurella canis in a soft tissue infection caused by a dog bite: the first report in Korea. Ann Lab Med. 2016;36(6):617‐619. 27578520 10.3343/alm.2016.36.6.617 PMC5011120

[6] Gundluru R , Bheemanathini P , Rafee Y . Pasteurella canis peritonitis in a child on peritoneal dialysis. Pediatr Infect Dis J. 2015;34(3):332. 25742087 10.1097/INF.0000000000000575

[7] Akahane T , Nagata M , Matsumoto T , et al. A case of wound dual infection with Pasteurella dagmatis and Pasteurella canis resulting from a dog bite – limitations of Vitek‐2 system in exact identification of Pasteurella species. Eur J Med Res. 2011;16(12):531‐536. 22112359 10.1186/2047-783X-16-12-531 PMC3351896

[8] Hara H , Ochiai T , Morishima T , Arashima Y , Kumasaka K , Kawano KY . Pasteurella canis osteomyelitis and cutaneous abscess after a domestic dog bite. J Am Acad Dermatol. 2002;46(5 Suppl):S151‐S152. 12004298 10.1067/mjd.2002.106350

[9] Ruan Z , Feng Y . BacWGSTdb, a database for genotyping and source tracking bacterial pathogens. Nucleic Acids Res. 2016;44(D1):D682‐D687. 26433226 10.1093/nar/gkv1004 PMC4702769

[10] Ruan Z , Yu Y , Feng Y . The global dissemination of bacterial infections necessitates the study of reverse genomic epidemiology. Brief Bioinform. 2019 10.1093/bib/bbz010 30715167

